# Non-Covalent Supported of l-Proline on Graphene Oxide/Fe_3_O_4_ Nanocomposite: A Novel, Highly Efficient and Superparamagnetically Separable Catalyst for the Synthesis of Bis-Pyrazole Derivatives

**DOI:** 10.3390/molecules23020330

**Published:** 2018-02-05

**Authors:** Mosadegh Keshavarz, Amanollah Zarei Ahmady, Luigi Vaccaro, Maryam Kardani

**Affiliations:** 1 Department of Gas and Petroleum, Yasouj University, Gachsaran 75918-74831, Iran; chem.mosadegh@gmail.com; 2 Nanotechnology Research Center, Ahvaz Jundishapur University of Medical Sciences, Ahvaz 14536-33143, Iran; 3 Department of Medicinal Chemistry, School of Pharmacy, Ahvaz Jundishapur University of Medical Sciences, Ahvaz 14536-33143, Iran; 4 Department of Chemistry, Biology and Biotechnology, University of Perugia, 06123 Perugia, Italy; luigi.vaccaro@unipg.it; 5 Marine Pharmaceutical Science Research Center, Ahvaz JundiShapur University of Medical sciences, Ahvaz 14536-33143, Iran; maryam138967@yahoo.com

**Keywords:** l-proline, nanocomposite, graphene oxide, heterogeneous catalyst, magnetically separable catalyst

## Abstract

A superparamagnetic graphene oxide/Fe_3_O_4_/l-proline nano hybrid that was obtained from the non-covalent immobilization of l-proline on graphene oxide/Fe_3_O_4_ nanocomposite was used as a new magnetically separable catalyst for the efficient synthesis of 4,4′-(arylmethylene)*bis*(1*H*-pyrazol-5-ol) derivatives. The prepared heterogeneous catalyst was characterized using FTIR, TGA, DTG, XRD, TEM, SEM, and elemental analysis techniques. Short reaction times (5–15 min), excellent yields (87–98%), and simple experimental procedure with an easy work-up are some of the advantages of the introduced catalyst.

## 1. Introduction

In recent years, carbocatalysis has attracted much attention, as it is cheap and can be easily obtained. Catalysts based on carbon constituents, such as activated carbon and carbon nanotubes, have also been used as catalyst supports [1]. More recently, graphene and graphene oxide (GO) are emerging as a novel class of carbocatalysts, as well as catalyst supports in organic synthesis [2,3]. GO has been proved as a favorable support due to its inherent properties, such as high chemically stability, large surface area, and good availability [4]. Most notably, GO has functional groups, mainly including epoxide, hydroxyl, and carboxyl on the edge, top, and bottom surface of each sheet that even after sufficient reduction, cannot be completely removed which makes GO a suitable support for metal and metal oxide nanoparticles [5]. Similar to carbon nanotubes, these functional groups can offer a platform for various chemical reactions. Recently, GO owing its surface decorated myriad oxygenated functions and conductivity with very high surface area has been used in sensors, nanoelectronics, contaminant removal, drug delivery, as metal-free catalysts or as supports for immobilizing active species for facilitating synthetic transformations. Among the various uses of GO, assembling other inorganic materials, like metal or metal oxide nanoparticles/nanocrystals on GO to fabricate composites or hybrids is an area of great potential [6,7,8,9]. Moreover, integrating GO with inorganic nanoparticles allows for the properties of the nanocomposite to be engineered for specific applications. In addition, the existence of the abundant oxygen functional groups affords GO sheets with great ability for the loading of organic molecules via covalent or non-covalent methods, simplifying the development of a broad new class of materials with improved properties, and even introducing new functionalities to GO sheet [10,11,12]. These catalysts have showed high catalytic activity and could be reused for several cycles.

Metal NPs can endow their unique properties to the resulting nanoassemblies derived from hybrid systems composed of polymer–nanoparticle or inorganic material–nanoparticle arrangements. The structure and functionalities of the host support can control the spatial distribution of nanoparticles. Therefore, the most prominent problem, which is the tendency of nanoparticles to agglomerate due to their high surface energy, can be avoided [13]. For example, various organic materials, such as dendrimers [14], polymers brushes [15], graphene sheets [16], as well as inorganic materials [17,18,19,20,21], have been utilized as carriers for the immobilization of metal NPs.

In recent years, amino acids, especially l-proline and proline-derivatives, have attracted much attention as promising catalysts for essential transformations in the fine chemical and pharmaceutical industries [22,23,24]. l-proline amino acid has been used more and more as catalyst, and is commercially available at low cost, but often employed at high catalyst loading as high as 30 mol % [25]. This unpleasant point is the main reason to take efforts for improving or modifying its catalytic activity through its immobilization on suitable supports, if and only the immobilization approach does not require the use of synthetic methodologies more expensive than proline itself [26].

When considering the above mentioned argument, we recently introduced a new facile and cheap methodology for the preparation of l-prolinate-amberlite adduct by using ion-pair immobilization of l-proline on the surface of commercially available amberlite IRA-900OH as a heterogeneous and recoverable organocatalyst for the efficient synthesis of 2-amino-4*H*-chromenes [27], spiroindoles [28], 3,3′-diaryloxindoles [29], bis-pyrazoles [30], 4*H*-pyrano[2,3-c] pyrazole derivatives [31], and biscoumarin derivatives [32].

Herein, we wish to introduce the l-proline on the GO/Fe_3_O_4_ nanocomposite through the non-covalent immobilization via hydrogen bonding interaction between the l-proline and the GO/Fe_3_O_4_ to prepare the GO/Fe_3_O_4_/l-proline nano hybrid. This technique gives robustness to the catalytic system, and, on the other hand, lets the l-proline organocatalyst to be flexible, mobile, and free on the surface of the GO/Fe_3_O_4_ support at the same time. Moreover, this method can enhance the thermal stability of the l-proline organocatalyst. Finally, this catalyst can be recovered simply by applying an external magnet. Such advantages are characteristic properties of homogeneous and heterogeneous catalysts, which have been included in GO/Fe_3_O_4_/l-proline hybrid. The prepared heterogeneous catalyst was used as an efficient and magnetically recoverable organocatalyst for the one-pot pseudo three-component synthesis of bis-pyrazoles in ethanol.

## 2. Results and Discussion

### 2.1. Catalyst Preparation

**Scheme 1:**
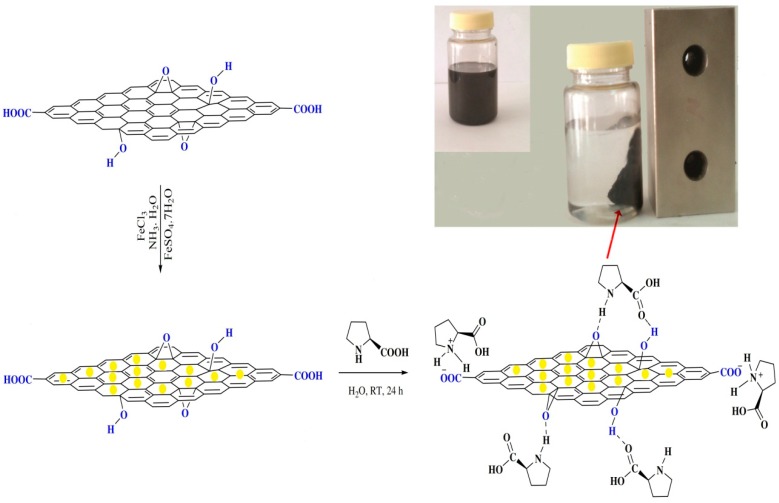
Preparation pathway of graphene oxide (GO)/Fe_3_O_4_/l-proline nano hybrid.

The concise method for the preparation of GO/Fe_3_O_4_/l-proline is illustrated in Scheme 1. GO and GO/Fe_3_O_4_ were prepared according to the modified Hummer’s method [33,34] and Song’s method [35], respectively. Then, a mixture of the achieved GO/Fe_3_O_4_ nano hybrid and pristine l-proline was sonicated in deionized water for 0.5 h and further stirred at room temperature for 24 h. Hydrogen-bonding interaction between hydroxyl, epoxy and carboxyl groups on the GO sheet in GO/Fe_3_O_4_ nano hybrid with carboxyl and secondary amine groups of l-proline is the driving force for l-proline binding to the GO/Fe_3_O_4_ nano hybrid, which has been presented in Scheme 1. The catalyst has been characterized by Fourier transform infrared (FT-IR), XRD, TGA, DTG, TEM, SEM, and elemental analysis.

### 2.2. Characterization of the Catalyst

#### 2.2.1. IR Spectra

**Figure 1:**
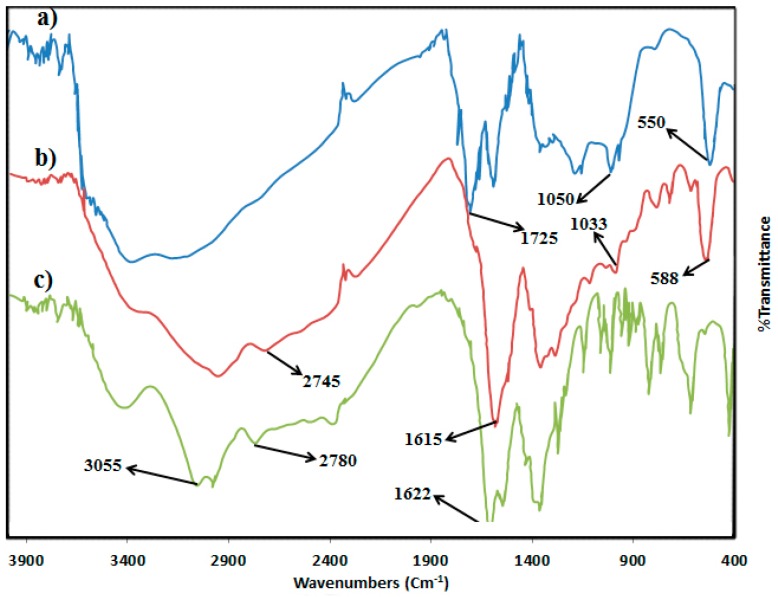
Fourier transform infrared (FT-IR) spectrum of GO (**a**), GO/Fe_3_O_4_/l-pro (**b**), and pristine l-proline (**c**).

The non-covalent immobilization of l-proline on the surface of GO/Fe_3_O_4_ nano hybrid can be confirmed by characterizing the pure GO, pristine l-proline, and GO/Fe_3_O_4_/l-proline nano hybrid using FT-IR spectroscopy, as shown in Figure 1. The FT-IR spectrum of GO displays distinguishing stretching vibration bands of carboxylic groups at round 3390, 1735, and 1224 cm^−1^, and stretching vibration band at 1050 cm^−1^ denotes to epoxy C-O groups (Figure 1a). The FT-IR spectrum of pristine l-proline shows typical stretching frequencies, including N-H asymmetric stretching at 3055 cm^−1^ and carboxylate (COO^−^) asymmetric and symmetric stretching at 1622 and 1380 cm^−1^, respectively (Figure 1c). These bands are appeared as new peaks in the FT-IR spectrum of GO/Fe_3_O_4_/l-proline nano hybrid when compared to the FT-IR spectrum of pure GO sheet (Figure 1a,b). The carboxylate (COO^−^) asymmetric and symmetric stretching of l-proline are presented in GO/Fe_3_O_4_/l-proline nano hybrid spectrum and seemed to move to lower positions at 1615 and 1375 cm^−1^, respectively (Figure 1b). The –Fe–O– stretching peak of GO/Fe_3_O_4_/l-proline appeared at 588 cm^−1^. Furthermore, the band at 3056 cm^−1^ corresponding to the asymmetric stretching vibration of the N-H group in l-proline is around 2800 cm^−1^ in FT-IR spectrum of GO/Fe_3_O_4_/l-proline. On the other hand, the wavenumbers of the characteristic bands of GO have also been decreased to lower locations and the individual band at 1735 cm^−1^ related to the carboxyl group in the spectrum of GO sheet vanished after l-proline was loaded. All of these results from the comparison of FT-IR spectra propose that l-proline organocatalyst has been successfully and effectively loaded on the surface of GO/Fe_3_O_4_ nano hybrid through hydrogen bonding interaction between carboxylic and secondary amine groups of l-proline with hydroxyl, carboxyl, and epoxy groups on the GO sheets of GO/Fe_3_O_4_ nano hybrid.

#### 2.2.2. TGA and DTG Analysis

**Figure 2:**
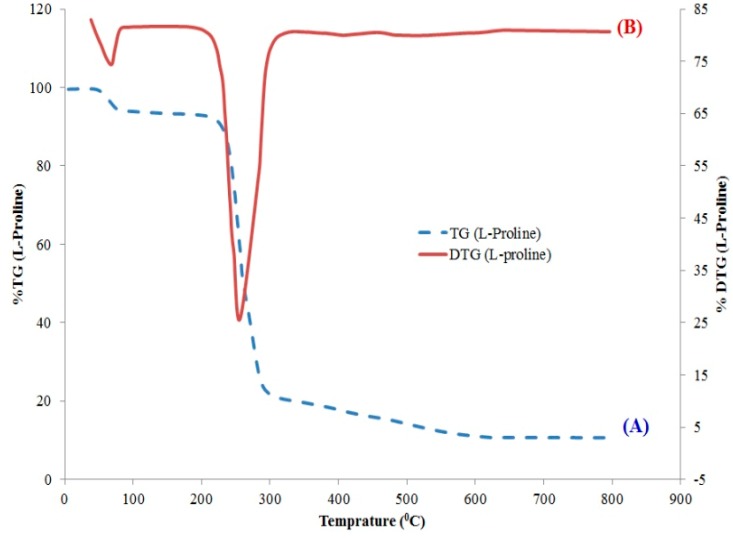
TGA (**A**) and DTG (**B**) curves of pristine l-proline.

**Figure 3:**
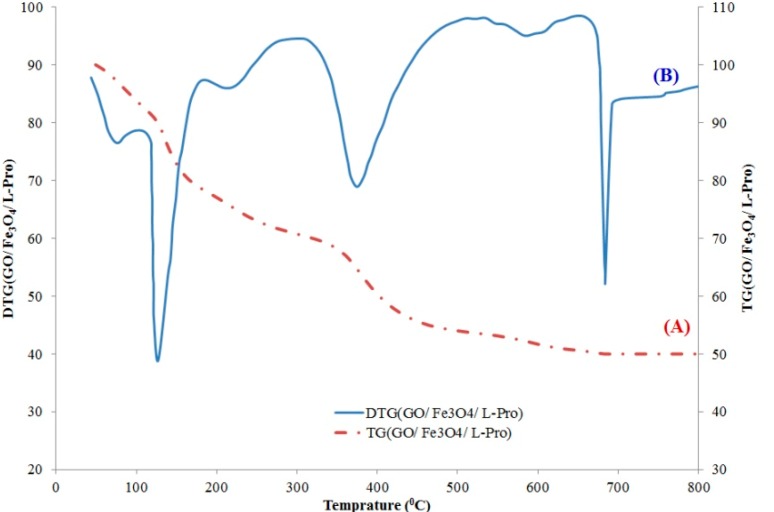
TGA (**A**) and DTG (**B**) curves of GO/Fe_3_O_4_/l-pro hybrid.

Thermogravimetric analysis (TGA) and differential thermal analysis (DTG) associated to the decomposition profiles of the pristine l-proline and GO/Fe_3_O_4_/l-proline nano hybrid under air atmosphere deliver additional proof for the immobilization of l-proline on the surfaces of GO/Fe_3_O_4_ nanocomposite (Figure 2 and Figure 3). The pristine l-proline shows two distinct steps of weight loss in the combined TG–DTG curves (Figure 2). The first weight loss centers around 60 °C, which is due to a loss of water. The second large weight loss assigned to the successive cleavage of the l-proline existed at 215–250 °C. The weight loss extends up to ca. 600 °C until the l-proline is almost entirely decomposed under air flow. Figure 3 illustrates the TG–DTG curves of GO/Fe_3_O_4_/l-proline nano hybrid. The first step (weight loss = ca. 10 wt %) before 100 °C is referred to the elimination of the surface-adsorbed water and interlayer water molecules. The second step contains the weight loss located at 125 °C and the trailed weight loss at 220 °C. The two weight loss peaks are assigned to the successive pyrolysis of the labile oxygen-containing functional groups (weight loss = ca. 20 wt %). This content of the highly rich oxygenated species is the main cause for the efficient loading of l-proline on the surface of GO/Fe_3_O_4_ nanocomposite. The third step, in the range of 310–750 °C (ca. 25 wt %), is reliable to refer to the successive cleavage of the l-proline moiety [36], since the weight loss in this range is nearly equal to the content of l-proline moiety calculated from the elemental analysis (24.5 wt %). The non-removable residue belongs to the remaining carbon and Fe_3_O_4_ nanoparticles. The increased decomposition temperature of the pristine l-proline from 215–250 °C to 310–750 °C range, when loaded on GO/Fe_3_O_4_ nano composite, suggests that the guest/host interaction was well done through the oxygen-containing functional groups of the GO sheet in GO/Fe_3_O_4_ support with carboxylic and secondary amine groups of l-proline (Scheme 1). This observation is also in accordance with the carboxylate asymmetric and symmetric stretching vibration of l-proline, which has been shifted to lower positions in FT-IR spectrum of GO/Fe_3_O_4_/l-proline (Figure 1b). Moreover, this increased thermal stability clearly confirms that the l-proline is successfully loaded on the GO/Fe_3_O_4_ support by forming the non-covalent hydrogen bond, rather than physical adsorption because this remarkable thermal stability could not be gained by physical adsorption. The high loading of l-proline on the surface of GO/Fe_3_O_4_ support (25 wt %), together with the unique non-covalent hydrogen binding behaviors between l-proline and GO/Fe_3_O_4_ support, makes the GO/Fe_3_O_4_/l-proline nano hybrid an efficient and stable catalyst in the reaction system.

#### 2.2.3. XRD

**Figure 4:**
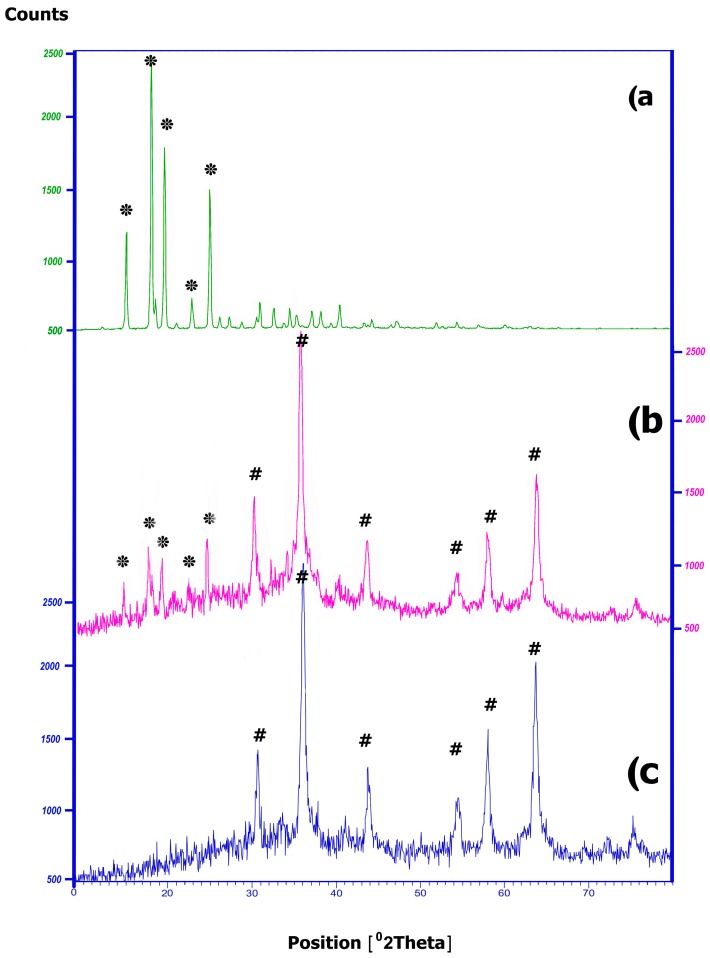
XRD patterns of pristine l-proline (**a**), GO/Fe_3_O_4_/l-pro (**b**), and GO/Fe_3_O_4_ (**c**). Symbles # and * refer to Fe_3_O_4_ NPs and L-proline patterns respectively.

Comparison of the XRD pattern of the synthesized GO with the XRD pattern of pure graghite indicated that the interlayer spacing between GO sheets is much more than graghite sheets (more than two times). Oxidation leads to the abundant formation of hydroxy, epoxy and carboxyl groups on the edge, top, and bottom surface the each sheet and increasing of the interlayer spacing between the GO sheets. This created interlayer spacing is a good opportunity for both Fe_3_O_4_ nanoparticles and l-proline organocatalyst to occupy interlayers and interact with GO functional groups, which is the cause that the loading amount of immobilized l-proline on GO is more than on graghite. The main peaks of pristine l-proline (denoted as *, 2θ) are shown in Figure 4a. Figure 4c displays the XRD pattern of GO/Fe_3_O_4_ support and the XRD pattern of GO/Fe_3_O_4_/l-proline hybrid is shown in Figure 4b. The main peaks (denoted as #, 2θ) in Figure 4c at 2θ = 30.1°, 35.6°, 43.6°, 53.6°, 57.5°, and 62.8° show the characteristics of Fe_3_O_4_ (JCPDS No. 19-629). The main intense diffraction peaks of pristine l-proline based on the standard spectrum are observed in the XRD pattern of GO/Fe_3_O_4_/l-proline hybrid due to the presence of the l-proline on the GO/Fe_3_O_4_ support (Figure 4a,b). The size of Fe_3_O_4_ nanoparticles was determined from X-ray line broadening using the Debye–Scherrer formula to be 25 nm.

#### 2.2.4. TEM and SEM

**Figure 5:**
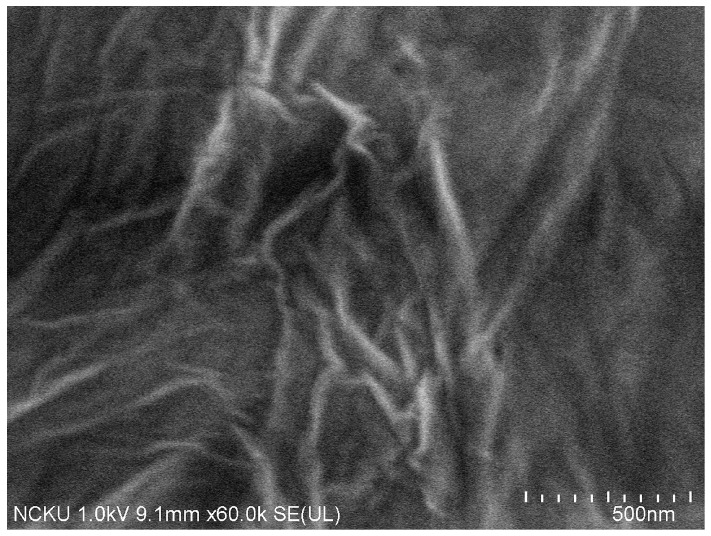
SEM image of synthesized graphene oxide.

**Figure 6:**
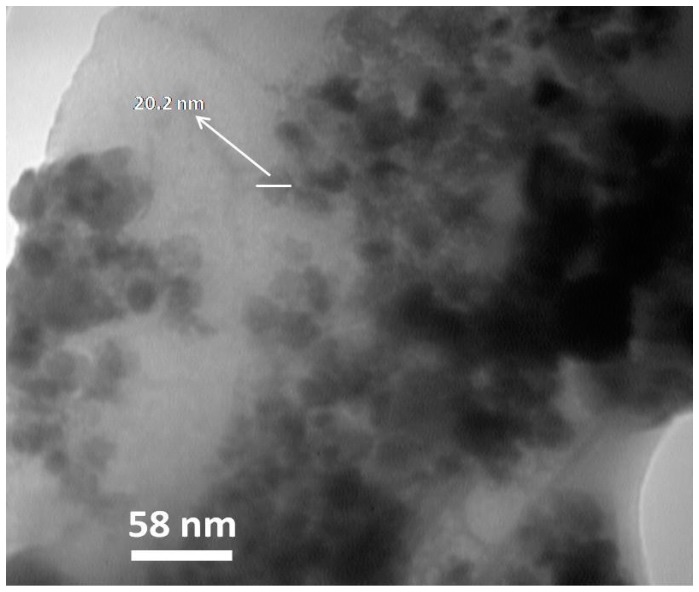
TEM image of fresh GO/Fe_3_O_4_/l-pro nano hybrid.

**Figure 7:**
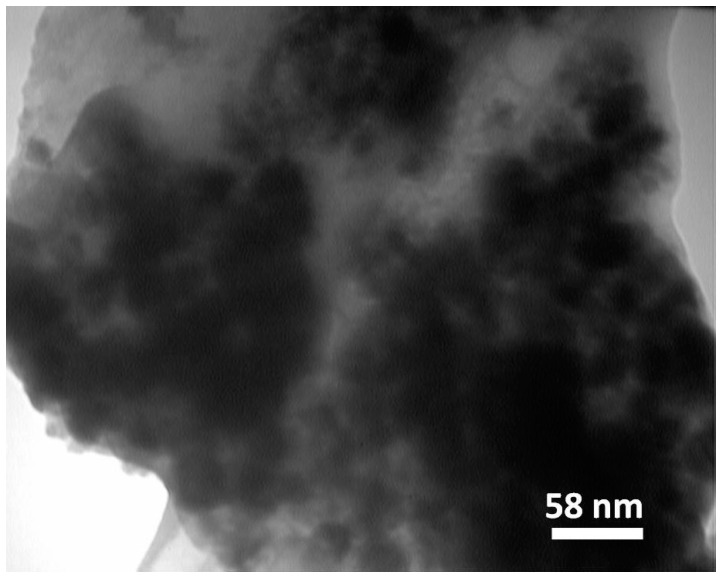
TEM image of reused GO/Fe_3_O_4_/l-pro nano hybrid after eight runs.

SEM was used to see the morphology of GO nanoplatelets. images of the prepared GO nanoplatelets show crumpled thin layers with wrinkles and folds on the surface of GO (Figure 5). The morphology of GO/Fe_3_O_4_/l-proline and the reused GO/Fe_3_O_4_/l-proline after eight runs was determined by TEM. As Figure 6 shows, Fe_3_O_4_ nanoparticles are chemically deposited on GO; although, some Fe_3_O_4_ aggregation is detected. The results of TEM confirmed that the nano-sized organic–inorganic hybrid material was prepared. The morphology of the reused catalyst did not show any significant change even after eight reaction cycles, which proved its robustness (Figure 7).

**Figure 8:**
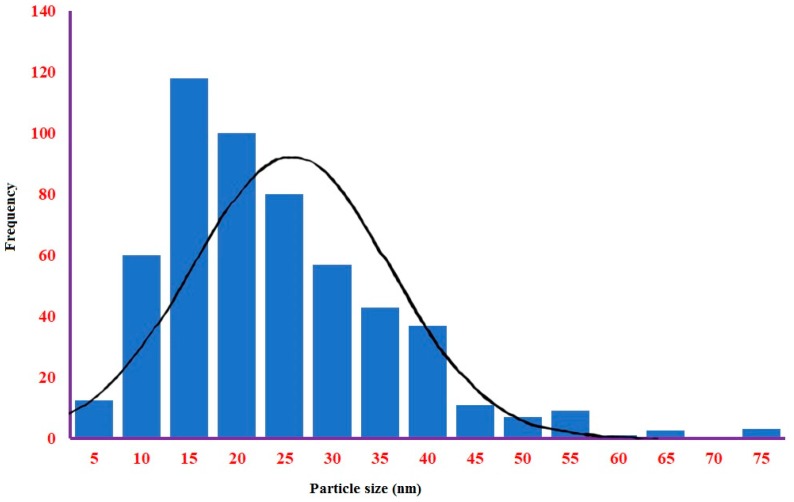
Number-frequency histogram of Fe_3_O_4_ nanoparticles in GO/Fe_3_O_4_/l-pro hybrid.

Size data of Fe_3_O_4_ nanoparticles were achieved by the counting of almost 500 particles from the TEM images of GO/Fe_3_O_4_/l-pro catalyst. Particle size and size distribution of Fe_3_O_4_ nanoparticles are represented in Figure 8. The number-frequency histogram illustrates the frequency of existence versus the size range of Fe_3_O_4_ nanoparticles. As it can be seen from Figure 8, the size distribution of the particles is twisted to the larger end of the particle-size scale. The mean size of Fe_3_O_4_ nanoparticles was obtained to be 24 nm with a standard deviation of 12.2.

### 2.3. Optimization of the Reaction Conditions

After the preparation and characterization of GO/Fe_3_O_4_/l-proline catalyst, its catalytic activity was investigated in a one-pot pseudo three-component reaction for the synthesis of 4,4′-(arylmethylene)bis(1*H*-pyrazol-5-ol) derivatives. To search for the optimal conditions, the reaction of benzaldehyde and two equivalents of 3-methyl-1-phenyl-2-pyrazolin-5-one was selected as the model reaction to examine the effect of GO/Fe_3_O_4_/l-proline catalyst (0.01–0.1 g), under a variety of conditions (Table 1).

The present optimization studies revealed that the best result was achieved by caring out the reaction in the presence of 0.05 g of GO/Fe_3_O_4_/l-proline under the reflux condition in ethanol (entry 10). The use of larger amounts of the catalyst (0.1 g, entry 11) did not improve the yield, while decreasing its amount led to decreased yield (Table 1, entries 8, 9).

**Scheme 2:**
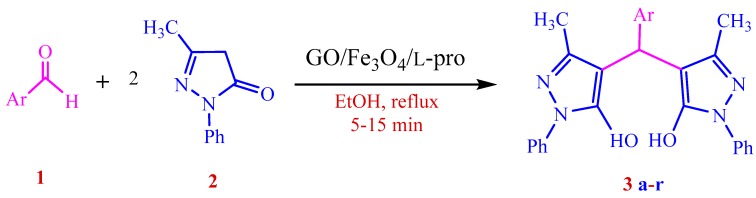
Synthesis of bis-pyrazole derivatives catalyzed by GO/Fe_3_O_4_/l-proline nano hybrid.

Using the optimized reaction conditions, the efficiency of this approach was explored for the synthesis of a wide variety of 4,4′-(arylmethylene)bis(1*H*-pyrazol-5-ol) derivatives (Scheme 2, Table 2). All of the reactions delivered excellent product yields and accommodated a wide range of aromatic aldehydes bearing both electron-donating and electron-withdrawing substituents (**3a**–**r**).

**Figure 9:**
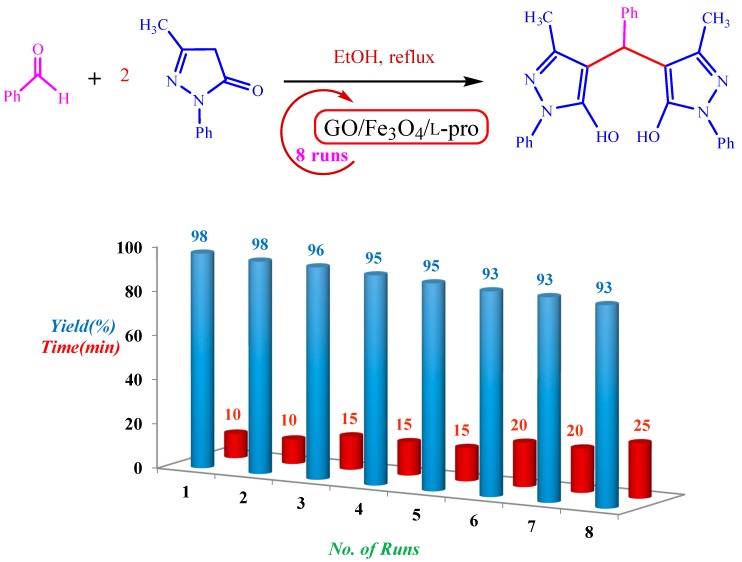
Recycling of GO/Fe_3_O_4_/l-pro nano hybrid for the synthesis of **3a**.

The recovery of a catalyst is highly preferred for a greener process. For this purpose, the reusability of GO/Fe_3_O_4_/l-proline was studied for eight consecutive cycles (fresh + seven cycles) for the synthesis of 4,4′-(phenylmethylene)bis(3-methyl-1-phenyl-1*H*-pyrazol-5-ol) (**3a**). From Figure 9, it can be observed that GO/Fe_3_O_4_/l-proline catalyst can be reused up to eight runs without the need to reload, and the yield difference between the first and eighth runs is only 5%, indicating that the catalyst efficiency is almost completely maintained during eight consecutive runs. The nitrogen content of the fresh and reused catalyst was measured using the elemental analysis and the comparison of the nitrogen contents suggested that the catalyst lost only 5% of its nitrogen content after eight runs. This is solid proof for the very low leaching account of l-proline organocatalyst from GO/Fe_3_O_4_/l-proline catalyst into the reaction mixture during eight runs, and also, confirms that the catalytic ability of GO/Fe_3_O_4_/l-proline has almost completely remained stable after eight runs, which is in agreement with the recyclability study. The TEM image of the recovered catalyst after eight runs did not reveal any significant change in the morphology of the reused catalyst, which proved its robustness while maintaining catalytic activity (Figure 7). Superparamagnetic properties and simple recovery of the catalyst are shown in Figure 9. GO/Fe_3_O_4_/l-proline catalyst can be recovered from the reaction mixture simply by applying an external magnet and its superparamagnetic properties remain constant even after eight runs.

**Figure 10:**
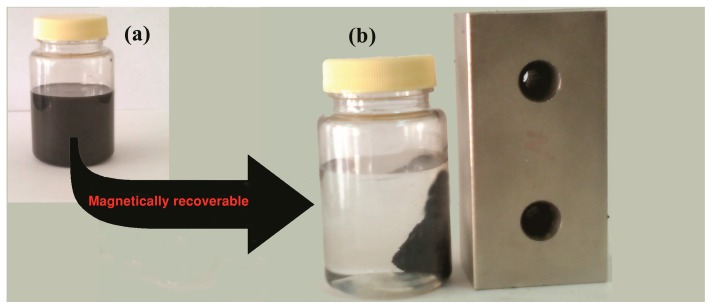
Powerful superparamagnetic property and simple recovery of GO/Fe_3_O_4_/l-proline catalyst using an external magnet.

**Figure 11:**
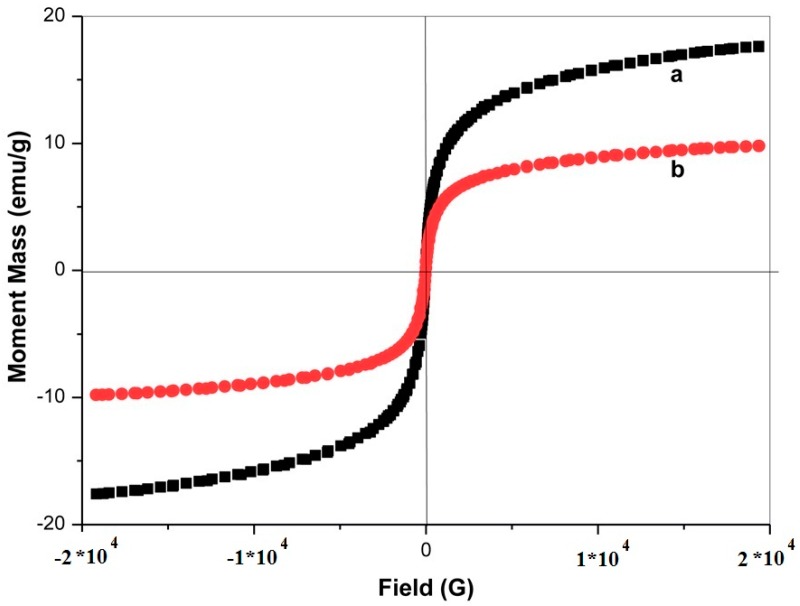
The magnetization curve of fresh GO/Fe_3_O_4_/l-pro (**a**) and reused GO/Fe_3_O_4_/l-pro after eight recycling (**b**).

Figure 10 shows the powerful superparamagnetic property of fresh GO/Fe_3_O_4_/l-pro by using an external magnet. For more detailed study, magnetic properties of fresh GO/Fe_3_O_4_/l-pro and the reused GO/Fe_3_O_4_/l-pro after eight runs were investigated using VSM (Figure 11). The achieved results illustrated that the catalyst samples have appropriate property for magnetic actuations, but there were some decrease in the values of saturation magnetization after eight consecutive runs, which possibly originated from the decrease of loading amount of Fe_3_O_4_ nanoparticles on the GO surface during using under eight repeated heating condition (Figure 11a,b).

It is worth noting that the introduced GO/Fe_3_O_4_/l-proline nano hybrid is a collection of three most applied catalysts, including GO, Fe_3_O_4_ nanoparticles, and pristine l-proline organocatalyst. Each of these catalysts has been separately used for several organic transformations. Hence, we became encouraged to compare the catalytic efficiency of GO/Fe_3_O_4_/l-proline nano hybrid with each of its component catalysts for the preparation of 4,4′-(arylmethylene)bis(1*H*-pyrazol-5-ol) derivatives. The reaction of benzaldehyde and two equivalents of 3-methyl-1-phenyl-2-pyrazolin-5-one was selected again as the model reaction (Table 3). On the base of the CHN analysis and TG (disscused above), the loading amount of l-proline in 0.05 g of GO/Fe_3_O_4_/l-proline nano hybrid is 0.01 g. To compare the catalytic activity of pristine l-proline with l-proline on GO/Fe_3_O_4_ nanocomposite, the equivalent amount of pristine l-proline (0.01 g) was also used as catalyst (entry **1** vs. **4**).

The results show that the l-proline immobilized on GO/Fe_3_O_4_ nanocomposite has better catalytic activity than pristine l-proline with the same loading amount of l-proline. This observation confirms that the catalytic activity of l-proline is improved when is immobilized on GO/Fe_3_O_4_ nanocomposite. Moreover, GO/Fe_3_O_4_/l-proline is a more powerful catalyst than its other component catalysts regarding the reaction time and the yield of the product (entry **1** vs. **2**, **3** and **5**). Finally, to show the efficiency of this method in comparison with other reported procedures, we selected the reaction of benzaldehyde and two equivalents of 3-methyl-1-phenyl-2-pyrazolin-5-one for the synthesis of 4,4′-(phenylmethylene)bis(3-methyl-1-phenyl-1*H*-pyrazol-5-ol) (**3a**) as a representative model. This comparison is shown in Table 4. It is clear from the data that our method has shorter reaction times and provides higher yields of the products.

## 3. Experimental

All of the chemicals were purchased from Merck and Aldrich. GO was prepared using modified Hummers method from flake graphite (Merck Company, Darmstadt, Germany). The reaction progresses were monitored by thin layer chromatography (TLC; silica-gel 60 F_254_, *n*-hexane: AcOEt). The ^1^H-NMR spectra were obtained on a Bruker Instrument DPX-400 Avance 2 model spectrometer (Bruker, Billerica, MA, USA). The vario El CHNS (made in Germany) was used for elemental analysis. Scanning electron micrographs (SEM) were obtained using a Cambridge S-360 instrument with an accelerating voltage of 20 kV (Cambridge, Austin, TX, USA). The powder X-ray diffraction (XRD) pattern was obtained by a Bruker AXS (D8, Avance) (Avance, San Antonio, TX, USA) instrument employing the reflection Bragg–Brentano geometry with CuKa radiation. A continuous scan mode was used to collect 2θ from 5° to 40°. Fourier transform infrared (FT-IR) spectra were obtained as potassium bromide pellets in the range 400–4000 cm^−1^ using an AVATAR 370 Thermo Nicolet spectrophotometer. The thermogravimetric and differential thermogravimetric (TG–DTG) analysis was performed on Netzsch STA449c (Kimia Sanat Ara, Tehran, Iran). The sample weight was ca. 10 mg and was heated from room temperature up to 600 °C with 10 °C/min using alumina sample holders. The structure of the products was confirmed on the basis of IR, NMR spectroscopic data, and elemental analysis.

### 3.1. General Procedure for the Preparation of GO

A flask containing graphite (1 g) and NaNO_3_ (0.75 g) were placed in an ice-water bath. H_2_SO_4_ (75 mL) was added with stirring, and then KMnO_4_ (4.5 g) was slowly added during about 1 h. After strongly stirring for 3 days at room temperature, 5% H_2_SO_4_ (140 mL) aqueous solution was slowly added over for about 1 h with stirring, keeping the temperature at 98 °C. Then, the temperature was reduced to 60 °C, 3 mL of H_2_O_2_ (30 wt % aqueous solution) was added, and the mixture was stirred for 2 h at room temperature. The prepared GO was suspended in pure water to give a brown dispersion, which was subjected to dialysis to absolutely remove the remaining salts and acids. The resultant purified GO powders were collected by centrifugation and air-dried [33,34].

### 3.2. Preparation of GO/Fe_3_O_4_ Nanocomposite

The GO/Fe_3_O_4_ nanocomposite was prepared by the chemical co-precipitation method similar to that of described above [35]. GO (200 mg) was dispersed into deionized water (250 mL) under ultrasonication for 30 min. FeCl_3_ (1.0 mmol) was added into the aqueous dispersion solution. The mixture was stirred overnight at 65 °C. Then, NH_3_·H_2_O solution (30 mL, 25–28%) and FeSO_4_·7H_2_O (0.5 mmol) were added, respectively. After the prepared mixture was stirred for additional 2 h at 65 °C, the precipitate was separated by an external permanent magnet, and it was washed with distilled water until neutralization and dried under vacuum.

### 3.3. Preparation of GO/Fe_3_O_4_/l-Proline Nano Hybrid

The mixture of the dried GO/Fe_3_O_4_ (1 g) and l-proline (0.5 g) was sonicated in deionized water for 0.5 h and further stirred at room temperature for 24 h. The prepared GO/Fe_3_O_4_/l-proline nano hybrid was separated by an external permanent magnet and dried under vacuum. The prepared catalyst was characterized using FTIR, TGA, DTG, XRD, TEM, and elemental analysis techniques. Based on TGA and elemental analysis the l-proline content of GO/Fe_3_O_4_/l-proline hybrid was obtained at 24–25 wt % (0.25 g of l-proline per 1 g of GO/Fe_3_O_4_/l-proline hybrid).

### 3.4. General Procedure for the GO/Fe_3_O_4_/l-Proline Nanohybrid Catalyzed Synthesis of 4,4′-(Arylmethylene)bis(1H-pyrazol-5-ol) Derivatives

A mixture of 3-methyl-1-phenyl-2-pyrazoline-5-one (2 mmol), aromatic aldehyde (1 mmol), were placed together in a round-bottom flask containing 5 mL of EtOH. Subsequently, GO/Fe_3_O_4_/l-proline nanohybrid catalyst (0.05 g) was added to the mixture. The suspension was magnetically stirred at reflux condition for sufficient time according to Table 2. After the completion of the reaction as followed by TLC (*n*-hexane:ethyl acetate; 3:1), the catalyst was separated by an external permanent magnet. The recovered catalyst was washed with acetone, dried, and stored for other similar repeated runs. The remaining mixture was filtered and cooled to room temperature to afford the pure crystals of 4,4′-(arylmethylene)bis(1*H*-pyrazol-5-ol) derivatives. The products are known compounds and their IR, NMR spectroscopy data, and melting points are compared with the reported values [30,37,38,39,40,41,42,43,44,45,46,47].

## 4. Conclusions

In summary, the introduced GO/Fe_3_O_4_/l-proline nano hybrid was prepared via hydrogen bonding interaction between the l-proline and GO/Fe_3_O_4_ nanocomposite. This technique gives robustness to the catalytic system; on the other hand, lets the l-proline organocatalyst to be flexible, mobile, and free on the surface of the GO/Fe_3_O_4_ support. Moreover, this method improves the thermal stability of the l-proline organocatalyst. The results showed that immobilized l-proline on GO/Fe_3_O_4_ nanocomposite has better catalytic activity than pristine l-prolin at the same wight ratios. GO/Fe_3_O_4_/l-proline catalyst can be recovered simply by applying an external magnet and reused at least for six runs for the one-pot pseudo three-component synthesis of 4,4′-(arylmethylene)bis(1*H*-pyrazol-5-ol) derivatives.

## Figures and Tables

**Table 1:**
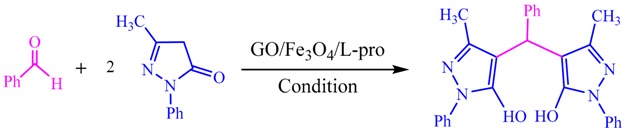
Investigation of catalytic activity of GO/Fe_3_O_4_/l-pro for the synthesis of -bis-pyrazole (**3a**) under various Conditions.

Entry	Conditions	Temperature (°C)	GO/Fe_3_O_4_/l-pro (g)	Time (min)	Yield (%) ^a^
1	neat	100	0.02	40	60
2	CH_2_Cl_2_	reflux	0.02	45	65
3	CH_3_CN	reflux	0.02	40	55
4	THF	65	0.02	45	60
5	DMF	100	0.02	45	65
6	H_2_O/DMF	100	0.02	20	75
7	H_2_O	reflux	0.02	25	80
8	CH_3_CH_2_OH	reflux	-	120	40
9	CH_3_CH_2_OH	reflux	0.02	20	92
10	CH_3_CH_2_OH	reflux	0.05	10	98
11	CH_3_CH_2_OH	reflux	0.1	10	95

^a^ Yield refer to isolated and pure product.

**Table 2:** Synthesis of bis-pyrazole derivatives (**3a**–**r**) catalyzed by GO/Fe_3_O_4_/l-proline.

Entry	Ar	Product	Time (min)	Yield ^a^ (%)	m.p. (°C)	
					Reported	Found
1	C_6_H_5_-	**3a**	10	98	171–172	170–172
2	4-Me-C_6_H_4_-	**3b**	13	94	202–204	203–205
3	4-Cl-C_6_H_4_-	**3c**	8	93	213–215	213–215
4	2-Cl-C_6_H_4_-	**3d**	10	91	236–237	233–235
5	2,4-(Cl)_2_-C_6_H_3_-	**3e**	10	94	227–229	227–229
6	4-NO_2_-C_6_H_4_-	**3f**	5	96	224–226	225–227
7	3-NO_2_-C_6_H_4_-	**3g**	8	90	149–150	152–154
8	4-OH-C_6_H_4_-	**3h**	15	92	152–153	153–155
9	3-OH-C_6_H_4_-	**3i**	14	87	165–168	169–170
10	3,4-(MeO)_2_-C_6_H_3_-	**3j**	7	90	195–197	194–196
11	4-MeS-C_6_H_4_-	**3k**	15	89	201–203	205–207
12	4-CN-C_6_H_4_-	**3l**	15	95	210–212	210–212
13	2-OH-C_6_H_4_-	**3m**	15	89	230–231	232–234
14	4-F-C_6_H_4_-	**3n**	13	93	180–182	180–182
15	4-*^i^*Pr-C_6_H_4_-	**3o**	12	87	132–134	132–134
16	4-MeO-C_6_H_4_-	**3p**	10	91	172–174	173–175
17	2-Br-C_6_H_4_-	**3q**	8	94	198–200	198–200
18	3-Br-C_6_H_4_-	**3r**	5	89	173–175	170–173

^a^ Isolated yields.

**Table 3:**
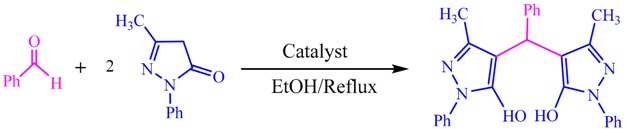
Catalytic activity of GO/Fe_3_O_4_/l-proline nano hybrid in comparison with pure GO, Fe_3_O_4_ nanoparticles, prestine l-proline, and GO/Fe_3_O_4_ nanocomposite.

Entry	Catalyst	Time (min)	Yield (%)
1	GO/Fe_3_O_4_/l-pro (0.05 g)	10	98
2	GO (0.05 g)	120	70
3	Fe_3_O_4_ nanoparticle (0.05 g)	90	80
4	l-Proline (0.01 g)	45	90
5	GO/Fe_3_O_4_ (0.05 g)	40	89

**Table 4:** Comparison of the catalytic efficiency of GO/Fe_3_O_4_/l-pro with various catalysts reported for the synthesis of **3a**.

Entry	Catalyst	Condition	Time (min)	Yield (%)	Ref.
1	GO/Fe_3_O_4_/l-pro (0.05 g)	EtOH/reflux	10	98	This work
2	[Amb]l-prolinate (10 mol %)	EtOH/reflux	11	97	[30]
3	NiFe_2_O_4_@SiO_2_–H_3_PW_12_O_40_	EtOH/reflux	15	91	[37]
4	Nano–SiO_2_/HClO_4_	H_2_O/reflux	20	94	[38]
5	SBNPTT	EtOH/reflux	30	90	[39]
6	[Pyridine–SO_3_H]Cl (1 mol %)	Solvent-free /50 °C	11	89	[40]
7	Phosphomolybdic acid	EtOH/r.t.	240	96	[41]
8	AP-SiO_2_ (30 mol %)	CH_3_CN/r.t.	10	98	[42]
9	PEG-SO_3_H (1.5 mol %)	H_2_O/reflux	30	92	[43]
10	LiOH·H_2_O (10 mol %)	H_2_O/90 °C	75	80	[44]
11	[Dsim]AlCl_4_ (1 mol %)	Solvent-free/50 °C	60	86	[45]
12	SASPSPE ( 0.1 g)	EtOH/reflux	18	90	[46]
13	SBSSA (18 mol %)	EtOH/reflux	120	80	[47]
